# Radiotherapy after the easing of public restrictions during COVID-19 epidemic

**DOI:** 10.1186/s13014-020-01612-5

**Published:** 2020-07-09

**Authors:** Weiping Wang, Ke Hu, Jie Qiu, Fuquan Zhang

**Affiliations:** Department of Radiation Oncology, Peking Union Medical College Hospital, Chinese Academy of Medical Sciences & Peking Union Medical College, No.1 Shuaifuyuan Wangfujing Dongcheng District, Beijing, 100730 China

**Keywords:** COVID-19, Radiotherapy, Reopen

## Abstract

The daily new confirmed Coronavirus disease 2019 (COVID-19) cases have decreased in some European and Asian countries. Many countries and areas have started to ease public restrictions. Here, we share our experiences and recommendations on how to conduct radiotherapy after public restrictions have eased or upon reopening. Firstly, COVID-19 nucleic acid test screening should be performed for all new admitted patients in areas with enough test capability. Secondly, radiotherapy can be conducted reference to consensus or recommendations on radiotherapy during COVID-19. Thirdly, it is not the time to consider compromising the guidance and the guidance on radiotherapy workflow and protection procedures still should be strictly followed.

## Main text

At present, the daily new confirmed cases and deaths due to Coronavirus disease 2019 (COVID-19) are still at a plateau worldwide. However, in some European and Asian countries, the daily new confirmed cases have dramatically decreased. The most challenging period has passed, and many countries or areas have started to ease public restrictions and reopen. Many eased or reopened areas still have considerable numbers of COVID-19 patients. After restrictions have eased and areas have reopened, there will be more patients in radiotherapy departments. The risk of clusters of severe acute respiratory syndrome coronavirus 2 (SARS-CoV-2) infection in radiation oncology departments may increase.

China was one of the earliest countries to ease public restrictions or reopen after the COVID-19 situation improved. After the patients with COVID-19 decreased to a low number, Wuhan, which was the centre of the COVID-19 pandemic in China, reopened on April 8, 2020. As the capital of China, Beijing also gradually eased public restrictions starting from the end of March. Peking Union Medical College Hospital is a comprehensive hospital with a fever clinic in Beijing. The hospital continued to conduct radiotherapy after the COVID-19 outbreak. Previously, we reported our experiences in the early stage of the COVID-19 pandemic [1]. Here, we share our experiences and recommendations on how to conduct radiotherapy after public restrictions have eased or upon reopening.

## COVID-19 nucleic acid test screening for all new admitted patients

In the early stage after the COVID-19 outbreak, the COVID-19 screening capability of most countries is limited, especially for nucleic acid testing. Two hospitals in Wuhan reported that all new admitted patients are receiving COVID-19 screening, including blood test, chest CT, and nucleic acid test, before radiotherapy [2, 3]. In most hospitals, including our institute, COVID-19 nucleic acid test screening was not conducted for patients without fever and other symptoms suspicious of COVID-19 [4–6]. In the past months, the COVID-19 nucleic acid test capability dramatically increased in most countries. It is reasonable to perform COVID-19 screening on all patients to decrease the risk of SARS-CoV-2 infection in the radiotherapy department. From May 6, we began to conduct COVID-19 nucleic acid testing for all new admitted patients in one week before radiotherapy. During the weeks of radiotherapy, nucleic acid testing is repeated for patients who get fever or have other symptoms suspicious of COVID-19. For patients with COVID-19, radiotherapy will be postponed.

## Reference to consensus or recommendations on radiotherapy during COVID-19

Patients with cancer are more vulnerable to SARS-CoV-2 [7]. During the COVID-19 pandemic, it was time to consider less as being better. Implementing hypofractionated radiotherapy schedules may decrease the access of patients to the hospital and limit the risk of SARS-CoV-2 infection [8]. In the past months, several radiotherapy consensus statements or recommendations have been published for lung cancer [9], rectal cancer [10], head and neck cancer [11], breast cancer [12], paediatric cancer [8], and interventional and intraoperative radiotherapy [13]. Compared with the early stage of the COVID-19 outbreak, there have been more consensus statements or recommendations to conduct radiotherapy. With these consensus statements or recommendations, radiation oncologists could conduct radiotherapy more appropriately during the COVID-19 outbreak. It should be noted that most consensus or recommendations are made in a comparatively short time, with limited discussion and evidence for some recommendations. And many of them recommend postponing or omitting radiotherapy in non-urgent cases. This might lead to undertreatment in many cases, so in the post-lockdown scenario, these recommendations need to be re-considered.

## Continue to follow the guidance on radiotherapy workflow and protection procedures

After the COVID-19 outbreak, there have been some reports on experiences or guidance on radiotherapy workflow and protection procedures for infection control zoning, area disinfection, personal protective equipment, staff rotation, COVID-19 screening, and emergence plans, among other measures [2–6]. The guidance should continue to be followed after the easing of restrictions or following reopening and should be gradually changed according to the COVID-19 situation in local areas. In our institute, patients and their escorts still must wear masks in radiotherapy area. Surgical masks are used by both medical and administrative staffs. Our institute restarted necessary face-to-face meetings for discussions of patients’ diseases and treatment from 11 May, 25 days after the last new confirmed COVID-19 case in Beijing. Academic meetings are still held online. In the case of full activity, it is difficult to conduct staff rotation. In this circumstance, our principle of scheduling is that, in one treatment room, the radiation therapists should be fixed. In this way, the range of infections that may be caused by potentially infected persons can be reduced. Other guidance is still strictly followed. For radiotherapy departments in most areas, it is not the time to consider compromising the guidance.

After 56 consecutive days without any new cases, a fresh cluster of cases was linked to a wholesale market in Beijing. From June 11 to 28, Beijing reported 318 confirmed cases. Thanks to the protective measure, no patient and medical staff have been infected with COVID-19 during the new outbreak in our institute.

## Data Availability

All data generated or analyzed during this study are included in this published article.

## Abbreviations

COVID-19: Coronavirus disease 2019
SARS-CoV-2: Severe acute respiratory syndrome coronavirus 2.
